# Supplementary material for: Timing of initiation of anti-retroviral therapy predicts post-treatment control of SIV replication
Source: PLoS Pathog. 2023 Oct 6;19(10):e1011660. doi: 10.1371/journal.ppat.1011660 (PMC10558076; doi:10.1371/journal.ppat.1011660)
Supplement: S1 Text — Summary of the experimental studies and additional analysis. (PDF) [file ppat.1011660.s001.pdf]

Study	Virus	Virus dose	Viral load detection threshold (c/ml)	Treatment			N	Monkey's IDs	Comments / setpoint time intervals	Known protective MHC-I alleles (A*01, B*08, B*17)	Reference
				Initiation	Regimen	Duration					
**National Cancer Institute (Cohort 1)**											
1.a	SIVmac239M (Barcoded)	$2.2x10^5$ IU (IV)	14	4	TFV, FTC, RAL, IDV, RTV	301	2	DEJX, DFGV.		All negative	(1)
1.a	SIVmac239M (Barcoded)	$2.2x10^5$ IU (IV)	14	4	TFV, FTC, RAL, IDV, RTV	370	2	DEJW, H090		All negative	(1)
1.a	SIVmac239M (Barcoded)	$2.2x10^5$ IU (IV)	14	4	TFV, FTC, RAL, IDV, RTV	478	2	DEPI, H105		All negative	(1)
1.b	SIVmac239M (Barcoded)	$2.2x10^5$ IU (IV)	14	6	TFV, FTC, RAL	81	3	MK9, KMB, KZ2		All A*01+	(1)
1.c	SIVmac239M (Barcoded)	$2.2x10^5$ IU (IV)	14	27	TFV, FTC, RAL, IDV, RTV	322	1	DEVW		Negative	(2)
1.c	SIVmac239M (Barcoded)	$2.2x10^5$ IU (IV)	14	27	TFV, FTC, RAL, IDV, RTV	369	2	DEVX, ZJ15		All negative	(2)
1.c	SIVmac239M (Barcoded)	$2.2x10^5$ IU (IV)	14	27	TFV, FTC, RAL, IDV, RTV	476	2	DEVJ, H106		All negative	(2)
1.d	SIVmac239X	$1.0x10^4$ IU (IR)	14	223	TDF, FTC, DTG	409	1	T028	Darunavir monotherapy started on day 124 and was maintained for the first 316 days of ART	Negative	
1.d	SIVmac239X	$1.0x10^3$ IU (IR)	14	356	TDF, FTC, DTG	410	2	T158, T159	Darunavir monotherapy started on day 202 or 213 and was maintained for the first 311 days of ART.	All A*01+	
1.d	SIVmac239X	$1.0x10^3$ IU (IR)	14	356	TDF, FTC, DTG	218	1	T156	Darunavir monotherapy started on day 213 and was	A*01+	

									maintained until the end of ART		
1.d	SIVmac239X	3.0x10^2 IU (IR)	14	377	TDF, FTC, DTG	410	1	T154	Darunavir monotherapy started on day 223 and was maintained for the first 311 days of ART.	A*01+	
**Oregon Regional Primate Center (Cohort 2)**											
2.a	SIVmac239X	2 FFU (IV)	30 or 1	6	TDF, FTC, DTG	605	13	24515, 24824, 26011, 28213, 23233, 27507, 25749, 27706, 27765, 28217, 23813, 25389, 26059	Vaccinated with CMV/SIV or control RhCMV. *The setpoint viral load at rebound in the subject 23813 was estimated between days 30 and 56.	23233 – B*17+; 25389 – B*08+, B*17+; 26059 – A*01+, B*17+; 24515 – B*08+, B*17+; 26011 – A*01+.	(3)
2.a	SIVmac239X	2 FFU (IV)	30 or 1	7	TDF, FTC, DTG	604	10	26470, 25420, 27812, 28232, 27826, 26475, 28092, 25280, 28473, 27046	Vaccinated with CMV/SIV or control RhCMV.	25420 – A*01+; 28232 – B*17+; 27046 – A*01+.	(3)
2.a	SIVmac239X	2 FFU (IV)	30 or 1	8-9	TDF, FTC, DTG	603, 602	4	25763, 22674, 22548, 28219	Vaccinated with CMV/SIV or control RhCMV.	25763 – A*01+; 28219 – A*01+, B*17+.	(3)
2.b	SIVmac239X	2 FFU (IV)	30 or 15 or 1	12	TDF, FTC, DTG	356	6	29258, 29277, 29529, 29710, 30649, 30709	Controls	All negative	(3)
2.c	SIVmac239X	2 FFU (IV)	30	42	TDF, FTC, DTG, DRV, RTV	928	18	27025, 27537, 27717, 27779, 27835, 27913, 27919, 28129, 28204, 28290, 28337, 28763, 27037, 27832, 27857, 27859, 28157, 28216	Vaccinated with CMV/SIV or control RhCMV. *The setpoint viral load in 14 out of 14 animals (with the available measurements after the day 30 post	27025 – A*01+; 27913 – A*01+; 28204 – A*01+; 27859 – A*01+, B*17+.	(3)

									detection of virus) in primary infection was estimated on the interval of 30 – 32 days post detection (shortest duration) and 30 – 38 (longest duration).		
**Emory National Primate Research Center (Cohort 3)**											
3.a	SIVmac239M (Barcoded)	$1x10^4$ IU (IV)	60	14	FTC, TDF, DTG	205/206	7	RLm14, RNd15, RYk16, 14C207, 9_047, REe16, RJy13	Controls only. *The setpoint viral load in 6 out of 6 animals (with the available measurements after the day 30 post detection of virus) after treatment interruption was estimated on the interval of 30 – 47 days post detection (shortest duration) and 30 – 53 (longest duration).	All A*01+.	Emory IACUC PROTO201 700655
3.b	SIVmac239	300 TCID50 (IV)	15	41	FTC, TDF, DTG	357	4	8R8, BV41, V304, 514	Controls only * The setpoint viral load in primary infection in all animals was estimated between days 30 and 34 post viral detection.	All negative	Emory IACUC PROTO201 700007 (4)
3.b	SIVmac239	300 TCID50 (IV)	15	43	FTC, TDF, DTG	357	2	V314, V309	Controls only. * The setpoint viral load in primary infection in all animals was estimated	All negative	Emory IACUC PROTO201 700007 (4)

									between days 30 and 36 post viral detection.		
3.c	SIVmac239	300 TCID50 (IV)	30	60	FTC, TDF, DTG	265-438	41	RHn16, RTj16, RAq16, RJr16, 138_14, RAi16, RVt16, RUm16, RVr16, RYi16, RYu16, RHs16, RZl16, RKt16, RZw15, RZn16, ROp16, RUv15, RFj16, RKi16, RQt16, RHk16, RIs16, RWn16, RLw16, RNi16, RQj16, RAg16, RGt16, RWs16, RPo16, RPn16, RFl16, RLt16, 82-13, RGi16, RJd16, 118-14, RTw16, RFu15, RHj16	Treated with immune checkpoint blockade (ICB), controls. 40 animals were followed up until rebound.<br><br>*The setpoint viral load in all animals in primary infection was estimated on the interval of 30 – 42 days post detection (shortest duration) and 30 – 51 (longest duration).	RKt16 – A*01+; RHn16 – A*01+; RGi16 – A*01+; RPo16 – A*01+; RZw15 – A*01+; RIs16 – A*01+; RWn16 – A*01+; RVr16 – A*01+.	(5)

Table A: Summary of the cohorts.

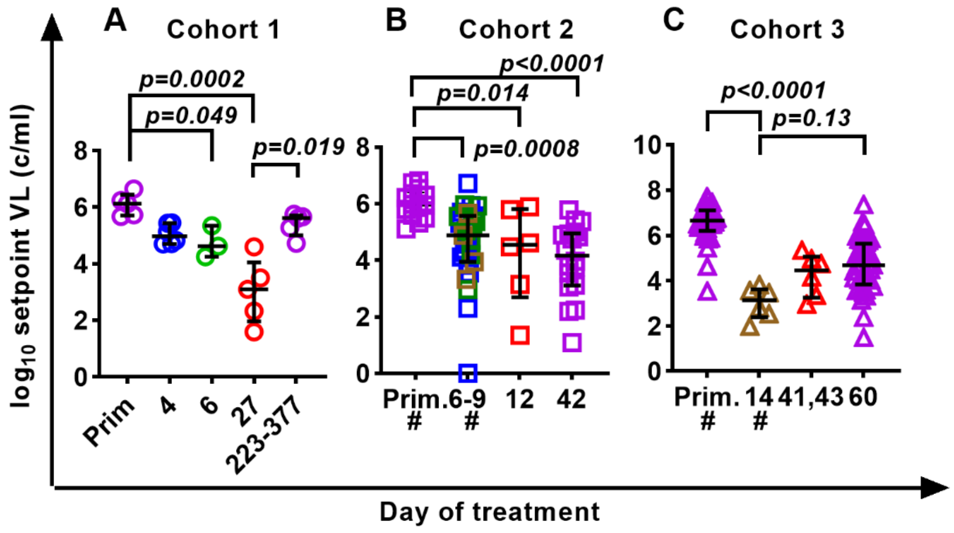

Fig. A: Dynamics of the post-rebound setpoint viral load broken down by cohort. # Time-weighted set-point viral loads were averaged over shorter time intervals for some animals. Descriptive statistics and statistical comparisons of the groups treated on different days are summarised in the tables below:

A. Cohort 1.

Descriptive Statistics					
**Statistic**	**Groups**				
	**Prim**	**4**	**6**	**27**	**223-377**
**Median**	6.131	4.983	4.626	3.104	5.618
**25% Percentile**	5.707	4.711	4.249	1.967	5.010
**75% Percentile**	6.447	5.445	5.349	4.054	5.714
**Mean**	6.088	5.046	4.741	3.029	5.413
**Std. Deviation**	0.3984	0.3456	0.5592	1.145	0.4201

Dunn's multiple comparisons test	
**Groups**	**Adjusted P Value**
**Prim vs. 6**	0.0490
**Prim vs. 27**	0.0002
**27 vs. 223-377**	0.0194
**Prim vs. 4**	0.0902

B. Cohort 2.

Descriptive Statistics				
**Statistic**	**Groups**			
	**Prim.**	**6-9**	**12**	**42**
**Median**	5.970	4.888	4.551	4.163
**25% Percentile**	5.577	3.946	2.698	3.112
**75% Percentile**	6.398	5.578	5.813	4.952
**Mean**	6.007	4.607	4.214	4.009
**Std. Deviation**	0.5151	1.379	1.723	1.280

Dunn's multiple comparisons test	
**Groups**	**Adjusted P Value**
**Prim. vs. 6-9**	0.0008
**Prim. vs. 12**	0.0138
**Prim. vs. 42**	<0.0001

C. Cohort 3.

Descriptive Statistics				
**Statistic**	**Groups**			
	Prim.	14	41,43	60
**Median**	6.660	3.142	4.455	4.678
**25% Percentile**	6.211	2.399	3.256	3.839
**75% Percentile**	7.106	3.626	5.065	5.644
**Mean**	6.556	3.032	4.257	4.693
**Std. Deviation**	0.7852	0.7077	0.9447	1.175

Dunn's multiple comparisons test	
**Groups**	**Adjusted P Value**
**14 vs. Prim.**	<0.0001
**14 vs. 41,43**	0.8986
**14 vs. 60**	0.1250

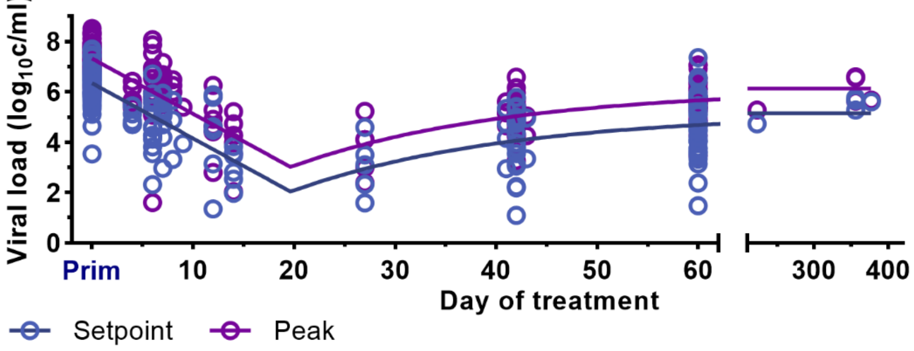

Fig B: The simultaneous fit of function (2) to $\log_{10}$ setpoint and peak viral load after rebound. Function (2) fitted to both setpoint and peak viral loads with the same parameters except for the maximal value, b_0 suggests that peak is approximately 10-fold higher than setpoint independent of the timing of ART initiation (shift on Y-axes is equal to 0.98 $\log_{10}$ c/ml).
Function (2) fitted with independent parameters for both datasets does not have a statistically better fit (F-Test's p-value = 0.91). The best-fit parameters for both models are in Table B

Model with the different parameters for each dataset						
**Dataset**		b_0	b_1	k_1	t_{min}	δ
**Setpoint (Fig 2B)**	**Best-fit values**	6.343	-0.2226	0.03675	18.53	-0.9484
	**Lower 95% conf. limit**	6.102	-0.2651	0.005544	13.57	-1.824
	**Upper 95% conf. limit**	6.584	-0.1801	0.06795	23.49	-0.9484
**Peaks (Fig 2C)**	**Best-fit values**	7.337	-0.216	0.06041	20.89	-1.405
	**Lower 95% conf. limit**	7.168	-0.247	0.01564	17.05	-1.960
	**Upper 95% conf. limit**	7.506	-0.1849	0.1052	24.73	-0.8492

Model with the same parameters (except b_0) for both datasets							
		b_0 (Setpoint)	b_0 (Peak)	b_1	k_1	t_{min}	δ
**Setpoint and peaks (Fig B)**	**Best-fit values**	6.352	7.330	-0.2192	0.0468	19.67	-1.192
	**Lower 95% conf. limit**	6.179	7.166	-0.2449	0.01988	16.51	-1.703
	**Upper 95% conf. limit**	6.525	7.493	-0.1935	0.07373	22.83	-0.6800

Table B: The best-fit parameters of the piecewise regression (equation (2)) fitted to the different datasets. The model with different parameters for each dataset does not fit better than the model where only b_0-s are different for setpoint and peak dataset, as determined by the F-test, p-value =0.91.

# of different parameters	Parameters that are different (all other parameters are shared)	AICc
1	$\boldsymbol{b_0}$	**-85.29**
1	b_1	-33.86
1	k	-14.96
1	δ	-13.83
1	t_{min}	-9.11
2	b_0, t_{min}	-83.31
2	b_0, δ	-83.48
2	b_0, b_1	-83.23
2	b_0, k	-83.24
2	b_1, t_{min}	-31.85
2	b_1, δ	-37.03
2	b_1, k	-34.44
2	δ , t_{min}	-13.36
2	δ, k	-13.90
2	k, t_{min}	-12.91
0	All parameters are the same	14.84
5	All parameters are different	-77.93

Table C: Corrected AIC for models that differ between datasets of setpoint and peak viral load by parameters shown. The model where the only different parameter is b_0 has better fit according to AICc (Fig B and Table B).

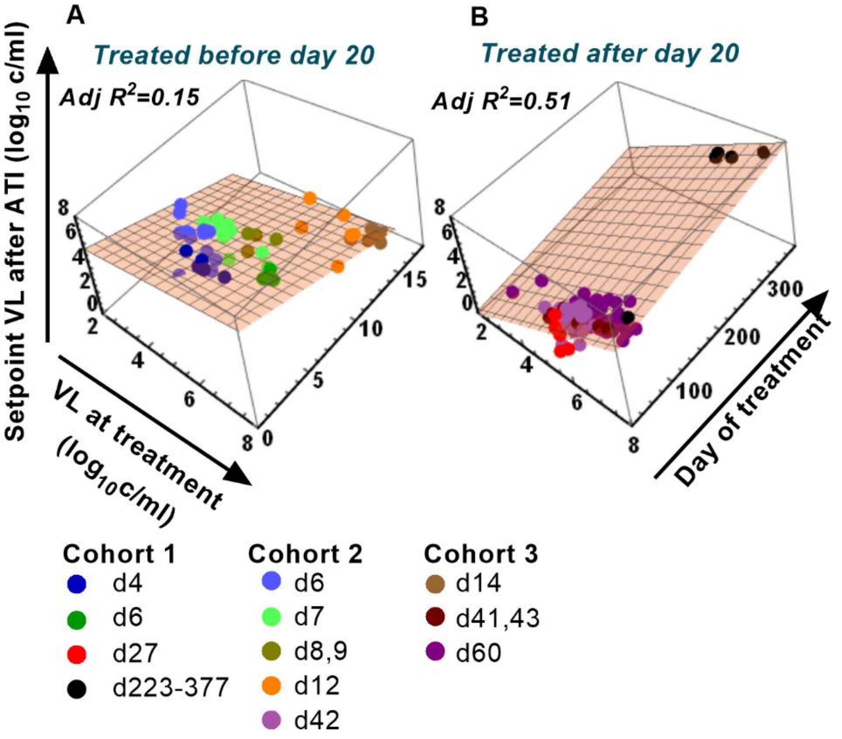

Fig C: Prediction of setpoint viral load using a 2-variable model.

A. For animals treated before day 20, timing of ART initiation is the strongest predictor of post-rebound setpoint VL (Fig 2D, main text). Adding data on the viral load at treatment initiation into the model did not significantly improve the fit (adjusted R^2=0.15 vs 0.13, p-value comparing model with day only and (day + VL) = 0.17).

B. For animals treated after day 20, viral load at treatment initiation is a good predictor of rebound setpoint viral load (Fig 2E, main text). Adding day of treatment as a factor significantly improves prediction (adjusted R^2=0.51 vs adjusted R^2=0.4, p-value comparing VL only with (VL + day) models <0.0001).

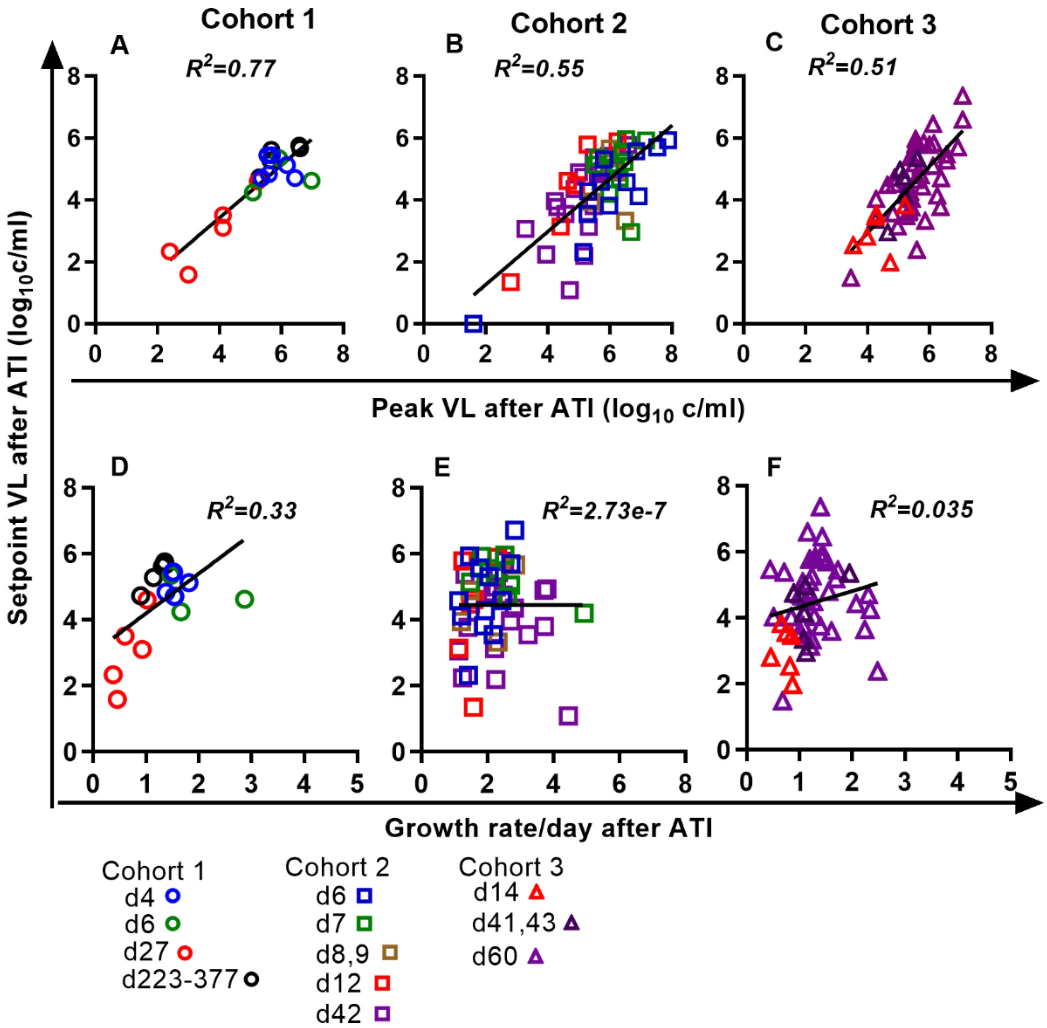

Fig D: Relationship between early post-rebound viral parameters and later setpoint viral loads.

A-C. Relationship between post-rebound peak and setpoint viral load.

	Correlation		Linear regression		
	**Spearman r**	**P value**	**Slope**	**Intercept**	**R squared**
**Cohort 1**	0.7433	0.0003	0.8524	0.01562	0.7741
**Cohort 2**	0.6485	<0.0001	0.8551	-0.4399	0.5461
**Cohort 3**	0.6732	<0.0001	1.075	-1.368	0.5123

D-F. Relationship between post-rebound viral growth rate and setpoint viral load.

	Correlation		Linear regression		
	**Spearman r**	**P value**	**Slope**	**Intercept**	**R squared**
**Cohort 1**	0.3351	0.1608	1.203	2.995	0.3337
**Cohort 2**	0.1562	0.2890	-0.0007501	4.453	2.725e-007
**Cohort 3**	0.3496	0.0111	0.4852	3.846	0.03469

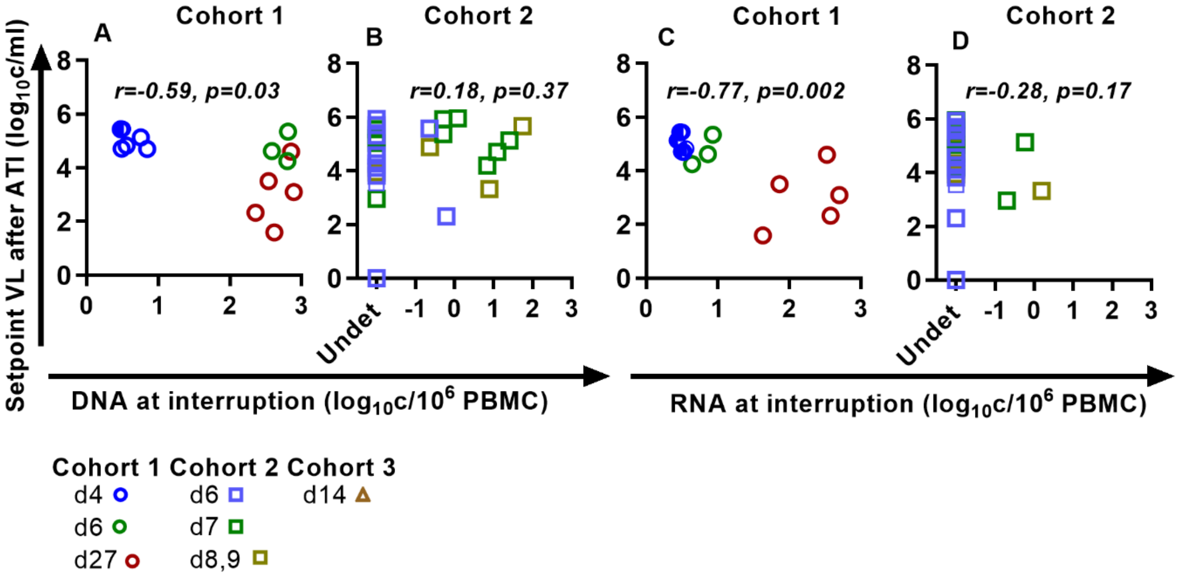

Fig E: Latent proviral reservoir by cohorts. (A, B) SIV DNA copies per million PBMC and (C, D) SIV RNA copies per million PBMC are negatively correlated with post-rebound control in groups from Cohort 1 (A, C) (Spearman r= -0.59, *p*=0.03 for DNA, and r=-0.77, *p*=0.002 for RNA), suggesting that larger reservoir size was associated with lower post-rebound setpoint viral load. However, no significant correlation was observed in data from Cohort 2 (B, D).

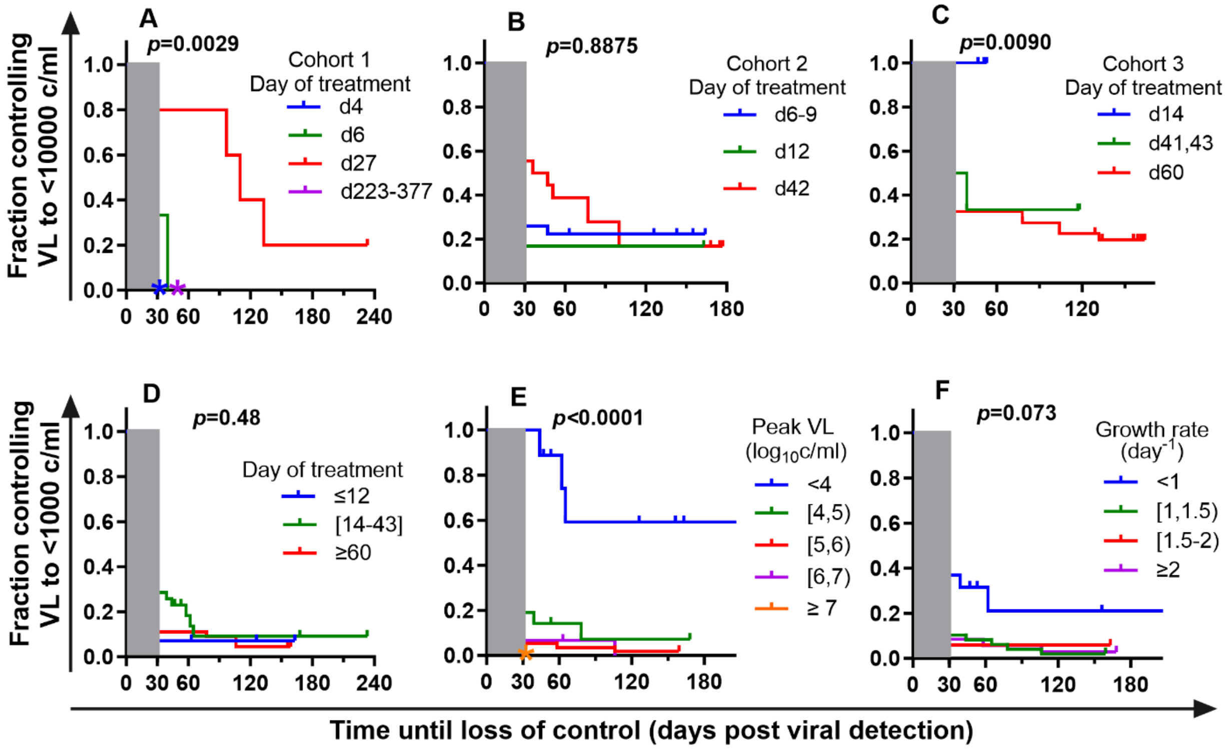

Fig F: Duration of post-rebound viral control. (A-C) The proportion of animals maintaining viral loads below 10,000 copies per ml over time post-rebound separated by cohort. The duration of control is significantly different between ART initiation groups in 2 of the 3 cohorts (p-values for the Log-rank test are shown in the figures).

(D-F) The Duration of post-rebound viral control below 1,000 copies per ml. (D) The proportion of animals maintaining viral control below 1,000 copies per ml is not significant (p-values for the Log-rank test are shown in the figures).

(E) Animals that have a low peak of the viral load during early rebound are more likely to maintain viral control below 1,000 copies per ml. (F) There is a trend for low viral growth rate during post-treatment rebound to be associated with longer-term control of post-rebound viral loads (not significant when considering four different levels of growth as shown, p = 0.073). However, comparing groups with the growth rate <1 and ≥1, we observed significant differences in the duration of control - p-value = 0.0085.

Coloured stars indicate groups where all animals had viral loads greater than 10,000 copies per ml (A) or 1,000 copies per ml (E) at day 30 post-detection. In order to avoid the initial post-rebound peak of viral load in the analysis of the duration of viral control, the first 30 days after detection of virus are ignored (shaded grey).

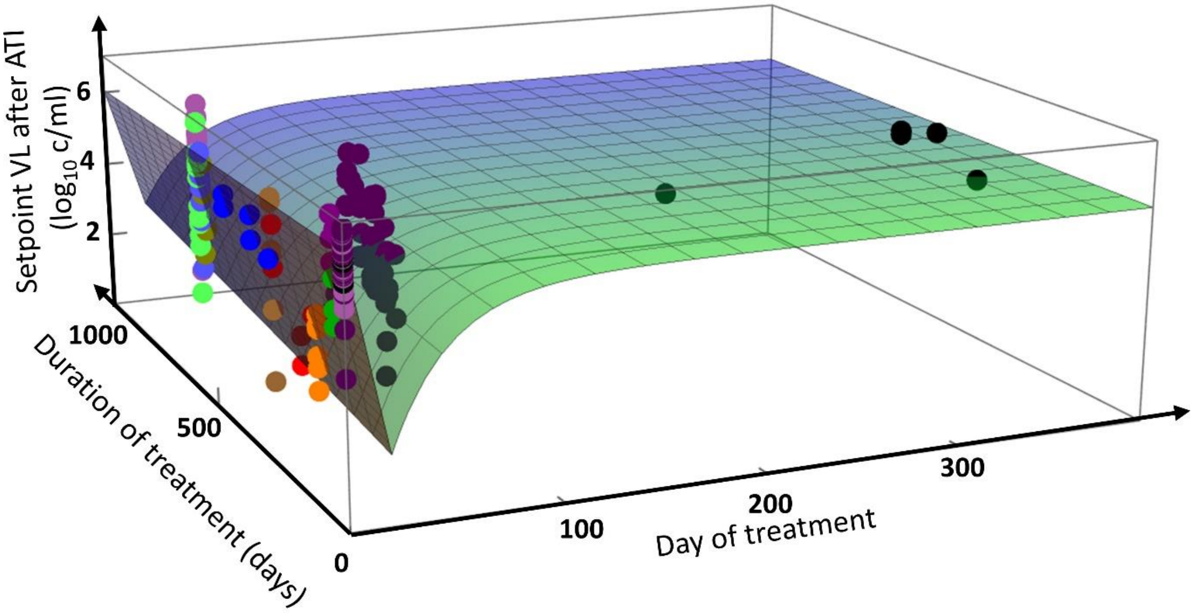

Fig G: Relationship between exposure to virus pre-treatment, duration of treatment, and post-rebound setpoint viral load according to the model defined by formula (3). Increasing exposure to virus before treatment leads to an initial decrease in post-rebound setpoint viral load (consistent with the priming of immune responses). However, further exposure to virus before treatment leads to increasing post-rebound setpoint viral load (consistent with immune exhaustion and/or viral escape). Prolonged treatment is associated with increased setpoint viral load post-rebound, which can be explained by the decline of immune memory and/or immune exhaustion due to exposure to low levels of viral antigen. We assume that setpoint at primary infection corresponds to the point when the day of treatment is equal to 0.

	Best fit parameters with 95% confidence intervals					
	b_0	b_1	k_1	t_{min}	δ	k_2
**Best-fit values**	6.351	-0.2383	0.0375	19.5813	-0.9827	0.0004
**Lower 95% conf. limit**	6.1064	-0.297	0.0073	14.0786	-1.865	-0.0004
**Upper 95% conf. limit**	6.5955	-0.1796	0.0676	25.084	-0.1005	0.0011

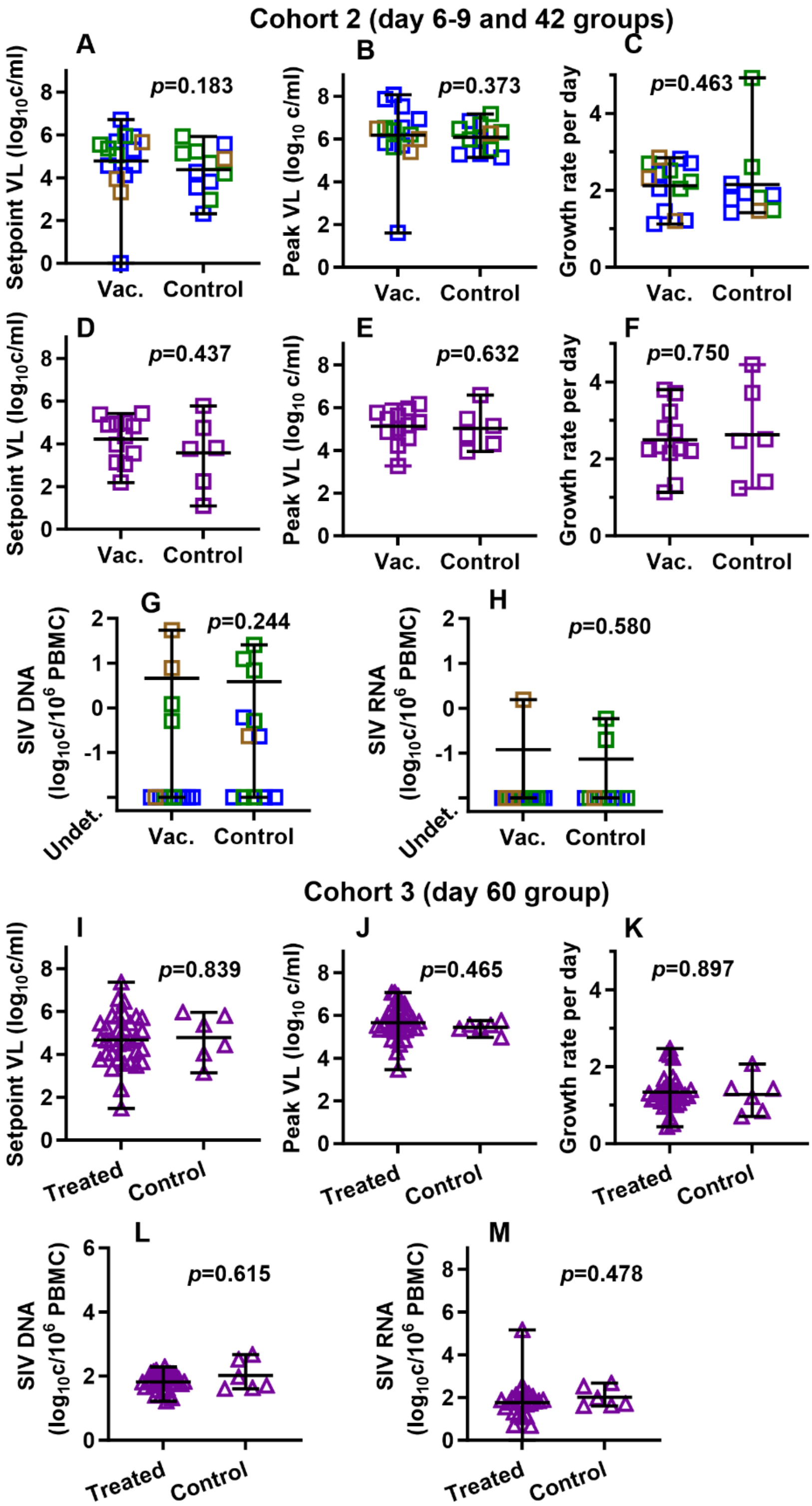

Fig H: No statistically significant difference in the studied parameters in vaccinated (cohort 2) or treated with immune checkpoint blockade (cohort 3) subgroups and control subgroups. Cohort 2, macaques treated with ART on days 6-9 and 42 (A-H) and cohort 3, macaques treated with ART on day 60 (I-M). No statistically significant difference between the median of vaccinated (cohort 2) or treated with ICB (cohort 3) and control subgroups by Mann-Whitney test with respect to the parameters discussed in the main text such as the rebound setpoint viral load (A, D, I), rebound peak viral load (B, E, J), rebound growth rate (C, F, K), SIV cell-associated DNA and RNA measured before ART interruption in cohort 2 (G, H) or on day 28 post-treatment in cohort 3 (L, M).

With the day 60 group.				
	With random effect for slope		Without random effect for slope	
	Intercept	Slope	Intercept	Slope
Fixed effect	0.32813740	0.01052793	0.03354471	0.01126580
Random effect (day 4)	0.7491399	0.010849268	0.6436764	na
Random effect (day 27)	-0.2618696	0.008771966	-1.2774460	na
Random effect (day 60)	0.4971419	0.011962558	0.7344037	na
p-value for parameter (F-test, anova() function)	<0.0001	2e-04	<0.0001	<0.0001
p-value for model comparison (likelihood ratio test, anova() function)	0.6807			
Without the day 60 group.				
	With random effect for slope		Without random effect for slope	
	Intercept	Slope	Intercept	Slope
Fixed effect	1.699270619	0.005972253	1.699234311	0.005972337
Random effect (day 4)	2.6909992	0.005972647	2.6911134	na
Random effect (day 27)	0.7075421	0.005971859	0.7073552	na
p-value for parameter (F-test, anova() function)	0.0013	0.1002	0.0013	0.1002
p-value for model comparison (likelihood ratio test, anova() function)	0.9759			

Table D. Best-fit parameter for the linear mixed effect model analysis of the relationship between the duration of treatment and the viral load setpoint at the rebound. A comparison of models with and without random effects for slopes shows that adding random effects does not improve the fit suggesting a similar rate of decay of protection among the groups.

	SIV DNA		SIV RNA	
	Intercept	Slope	Intercept	Slope
Fixed effect	4.4318997	-0.0666468	4.2819562	-0.2016088
Random effect (cohort 1)	4.431895	-0.06671734	4.281987	-0.2015914
Random effect (cohort 2)	4.431905	-0.06657626	4.281925	-0.2015763
p-value	<0.0001	0.5618	<0.0001	0.117

Table E. Best-fit parameter for the linear mixed effect model analysis of the relationship between the duration of treatment and the viral load setpoint at the rebound.

Dataset		Best fit parameters with 95% confidence intervals					
		b_0 protective		b_1	k_1	t_{min}	δ
		+	−				
Setpoints (Fig 8A)	Best-fit values	5.846	6.495	-0.2069	0.02910	19.46	-0.5887
	Lower 95% conf. limit	5.502	6.250	-0.2486	0.00922	14.95	-1.477
	Upper 95% conf. limit	6.190	6.739	-0.1653	0.04898	23.97	0.000
Peaks	Best-fit values	7.283	7.350	-0.2140	0.05884	20.96	-1.380
	Lower 95% conf. limit	7.015	7.173	-0.2460	0.01532	17.14	-1.954
	Upper 95% conf. limit	7.551	7.527	-0.1820	0.1024	24.79	-0.8056

Table F. The best-fit parameters of the piecewise regression defined by formula (2). The regression is fitted to the setpoint and peak viral loads according to the time of treatment and the presence or absence of protective MHC-class 1 alleles. The model in this table has different b_0 for the different groups, with all other parameters the same between groups. The parameters of the model with the same b_0 are in Table B.

Number of parameters that differ between datasets	Parameters that are different for datasets with presence or absence of protective MHC-class 1	AICc. Setpoint data	AICc. Peak data
1	b_0	-6.716	-91.46
1	b_1	1.486	-92.68
1	k	1.683	-91.21
1	δ	2.978	-91.21
1	t_{min}	6.46	-91.25
2	b_0, t_{min}	-4.948	-89.56
2	b_0, δ	-4.576	-89.33
2	b_0, b_1	-4.581	-90.64
2	b_0, k	-4.877	-89.32
2	b_1, t_{min}	5.138	-91.11
2	b_1, δ	1.994	-90.56
2	b_1, k	2.003	-90.67
2	δ , t_{min}	5.056	-89.26
2	δ, k	3.792	-89.04
2	k, t_{min}	0.1138	-90.17
0	All parameters are the same	5.927	-93.34
5	All parameters are different	-4.48	-85.73

Table G: Corrected AIC for models that differ by parameter shown between setpoint and peak viral load datasets with presence or absence of protective MHC-class 1 alleles. The model with b_0 as the only different parameter has better fit when fitting to setpoint viral loads, however for the peak viral load the best-fit model has the same parameters for datasets with the presence or absence of protective MHC-class 1 alleles.

Parameter	Correlation with post-rebound setpoint viral load	p-value	Test
Viral load at treatment initiation	Adjusted R^2= 0.040	p=0.016	Linear regression. F-test
Peak viral load in primary infection (all cohorts)	Adjusted R^2= -0.008	p=0.79	Linear regression. F-test.
Viral load at treatment initiation (treated <day 20 post-infection)	Adjusted R^2 = 0.0016	p=0.31	Linear regression. F-test.
Day of treatment (treated < day 20)	Adjusted R^2 = 0.13	p=0.0077	Linear regression. F-test.
Day of treatment and viral load at treatment initiation	Adjusted R^2=0.15	p=0.16	Multivariate linear regression. F-test of the day of treatment and viral load at treatment initiation vs. day of treatment
Viral load at treatment initiation (treated >day 20 post-infection)	Adjusted R^2 = 0.40	p <0.0001	Linear regression. F-test.
Peak viral load in primary infection (treated>day 20 post-infection)	Adjusted R^2 = 0.11	p=0.0024	Linear regression. F-test.
Duration of treatment (day 4, day 27 and day 60 groups)	Fixed effect slope =0.011	p<0.0001	Linear mixed effect model. t-test.
Duration of treatment (day 4, day 27)	Fixed effect slope =0.006	p=0.1	Linear mixed effect model. t-test.
Frequency of reactivation (cohort 1 - d4, 27; Cohort 3 – d14)	r=-0.58	p=0.016	Spearman coefficient of correlation
DNA at interruption (Cohort 1 - d4, d6, d27 and Cohort 2 – d6, d7, d8-9)	Fixed effect slope=-0.066	p=0.56	Linear mixed effect model. t-test.
RNA at interruption (Cohort 1 - d4, d6, d27 and Cohort 2 – d6, d7, d8-9)	Fixed effect slope=-0.202	p=0.117	Linear mixed effect model. t-test.
DNA at interruption (Cohort 1 - d4, d6, d27)	r=-0.59	p=0.03	Spearman coefficient of correlation
DNA at interruption (Cohort 2 – d6, d7, d8-9)	r=-0.18	p=0.37	Spearman coefficient of correlation
RNA at interruption (cohort 1- d4, d6, d27)	r=-0.77	p=0.002	Spearman coefficient of correlation
RNA at interruption (Cohort 2 – d6, d7, d8-9)	r=-0.28	p=0.17	Spearman coefficient of correlation
Time to detection (Cohort 1)	N/A	p=0.83	Log-rank (Mantel-Cox) test
Time to detection (Cohort 2)	N/A	p=0.56	Log-rank (Mantel-Cox) test
Time to detection (Cohort 3)	N/A	p=0.13	Log-rank (Mantel-Cox) test
CD107a (Cohort 1, d4, d27)	r=-0.82	p=0.0031	Spearman coefficient of correlation
Total CD8+ Response (Cohort 2, d6, d7, d8-9 Control)	r=0.08	p=0.80	Spearman coefficient of correlation
Total CD8+ Response (Cohort 2, d6, d7, d8-9 Vaccinated)	r=-0.22	p=0.42	Spearman coefficient of correlation

Table H: Summary of correlation analysis of parameters discussed in the main text of the manuscript.

Supplementary method.

Estimation of the duration of control

In order to estimate the duration of control, i.e. duration of VL below a nominated control threshold (CT), we ignore the initial 30 days post detection of virus (let us call the time after these 30 days a post-peak time - PPT) and we do not count these 30 days toward the estimate of the duration of control. The definition of the duration of control is based on 6 possible scenarios. The first three scenarios concern animals with at least two VL measurements during PPT:

1) We consider that the control is lost if two consecutive measurements during PPT are above the CT of 4 log_{10} copies/ml. We define the loss of control to be at the first of the two measurements above CT, and the duration of control = first measurement above CT – 30. If the first and the second measurements during PPT are above CT, then the duration of control is defined as 0.
2) If the viral load does not exceed the threshold for two consecutive measurements during PPT and the last follow up time point is below CT, then we consider that control is not lost until the last measurement (inclusively) and we censor the subject at the last time point.
3) If there is no more than one consecutive measurement above CT during PPT and the last measurement is above the detection threshold, then we censor the animal at the second last point (as we cannot tell if control is lost at the last point).

Now, let us consider the cases when there are less than two points during PPT so we cannot apply the above criteria directly.

4) If there is only one measurement during PPT and the VL is less than CT, then we censor the subject at this point.
5) If there is only one measurement during PPT and the VL is greater than CT, then the subject is excluded from the study (as we cannot confirm if this measurement would have been followed by a second reading above the threshold).
6) The subject is also excluded from the study if the follow-up terminates before PPT.

References

1. C. M. Fennessey *et al.*, Genetically-barcoded SIV facilitates enumeration of rebound variants and estimation of reactivation rates in nonhuman primates following interruption of suppressive antiretroviral therapy. *PLoS Pathog.* **13**, e1006359 (2017).
2. M. Pinkevych *et al.*, Predictors of SIV recrudescence following antiretroviral treatment interruption. *Elife* **8**, e49022 (2019).
3. A. A. Okoye *et al.*, Early antiretroviral therapy limits SIV reservoir establishment to delay or prevent post-treatment viral rebound. *Nat. Med.* **24**, 1430-1440 (2018).
4. M. Pino *et al.*, Limited impact of fingolimod treatment during the initial weeks of ART in SIV-infected rhesus macaques. *Nat. Commun.* **13**, 5055 (2022).
5. J. Harper *et al.*, CTLA-4 and PD-1 dual blockade induces SIV reactivation without control of rebound after antiretroviral therapy interruption. *Nat. Med.* **26**, 519-528 (2020).
