# Supplementary material for: Timing of initiation of anti-retroviral therapy predicts post-treatment control of SIV replication
Source: PLoS Pathog. 2023 Oct 6;19(10):e1011660. doi: 10.1371/journal.ppat.1011660 (PMC10558076; doi:10.1371/journal.ppat.1011660)
Supplement: Attachment — Submitted filename: Response 2023.06.20.docx [file ppat.1011660.s002.docx]

**Response to Reviewer's Questions**

**Part I - Summary**

**Reviewer #1**: Pinkevych and coworkers have addressed the issue of identifying determinants and predictors of SIV control after interruption of anti-retroviral therapy. These questions are important ones for understanding how viral control off therapy can be enhanced and sustained. They pooled data from different institutions and 10 different animal cohorts that comprised 124 SIV-infected/ ART-treated rhesus macaques that underwent a subsequent ATI. All animals were infected with SIVmac239 (± barcodes), although there were differences in the inoculating dose, route of infection, duration of treatment, and experimental designs of the pooled studies. Nonetheless, from this meta analysis, themes emerged in that 1) when treatment was given before 3 weeks of infection, the longer the time to initiate treatment, the lower the post ATI peak and set point; and 2) when treatment was started after 3 weeks of infection, the longer time to initiate treatment correlated with higher post-ATI set points. They argue that these different outcomes, dependent on the time of ART initiation, likely result from a maturation of immune responses during the first 3 weeks (i.e. the longer the better for post ATI-control) whereas after 3 weeks, these immune responses are not operative, likely due to immune exhaustion (i.e. the longer the time for starting therapy, the worse for post ATI-control).

As the authors note, NHP models offer advantages for addressing viral dynamics post ATI, and set points are rarely established given the medical need and ethics for ART to be re-initiated in humans.

It is a challenging undertaking to bring together data from multiple NHP studies from diverse experimental protocols, designed to address different questions. Although the trends the authors have identified are intriguing, and their hypothesis that early functional and later dysfunctional immune responses underlie their findings, there are alternative explanations and aspects that are not easily controlled for in this analysis of pooled data. These confounding issues are noted throughout the manuscript, and include the presence of controlling MHC alleles in animal cohorts, the timing and nature of ART used, and the extent to which viral escape is occurring.

We thank the reviewer for these supportive comments. We agree that a clear description of the potential confounders is very important, particularly in future interpretation of this in the context of other studies (where single studies may be more or less affected by confounders). We have attempted to discuss confounding issues as much as possible.

**Reviewer #2:** This work is very interesting and highly relevant regarding reservoir size before treatment initiation and viral load rebound after treatment interruption. The authors fit a simple model to viral load data of three different cohorts to analyze rebound post-treatment interruption and the association between pre-treatment peak viral load, the timing of treatment, and viral set point post-treatment interruption. The authors found time-dependent post-treatment control and set point viral load, where the viral load was better controlled when treatment was initiated within the first three weeks post-infection. Treatment initiation after three weeks led to higher post-rebound set point viral levels. Furthermore, the authors state that the peak viral load at treatment initiation is a primary predictor for post-treatment control and set point viral load.

I have some comments and suggestions regarding the manuscript.
The part about data explanation is excellent. However, the part about model analysis takes much work to follow. The modeling part of the manuscript is not reproducible in its current form. Furthermore, most model interpretations are based on weak associations, and the different results should be put into context with each other. The different results about treatment timing are very confusing. Please see the following comments.

We thank the reviewer for these positive and constructive comments. We are pleased they found the data explanation clear and apologise if they found the model description less clear. We have expanded these sections in response to the specific comments below. We also note that the full data and code will be made available on GitHub upon publication. We believe that together this should ensure that the work is now fully reproducible by others.

**Part II – Major Issues: Key Experiments Required for Acceptance**

**Reviewer #1:**

1. Beyond the data in Figure 1 showing the relationship of viral set point (VSP) and peak after ATI and time of ART initiation, many of the points discussed in groups and subgroups are complicated, and correlations or lack of correlations are difficult to tease out. For example, set point after rebound is later divided into early set point (reduced with early Rx) vs later set point. It seems that a Summary Table would be helpful with categories of
   Time to viral rebound
   VSP post rebound
   Peak post rebound
   Control post rebound
   vs. correlates or non-correlates listed for
   Early initiation of Rx
   Late initiation of Rx
   Duration of ART
   Viral Peak during primary infection
   Cell-associated DNA and RNA
   CD8 T cell responses

We apologise that the reviewer found this unclear and thank the reviewer for the suggestion of a summary table. We have now included this as Supplementary Table 8 in S1 Text as suggested, and also mentioned it in the text of the manuscript at lines 219-221.

*“These correlation analyses, as well as those of other parameters of interest from the Results section, are summarised in the Supplementary Table 8 in S1 Text)”*

2. The issue of controlling MHC alleles, which could be a major confounder in this analysis, was, surprisingly, not mentioned in the Results section. While this was discussed in the Discussion with data in a Supplemental Figure, it should be presented in Results and with data shown as a Figure. This reviewer still has concerns that effects of controlling or non-controlling alleles could be contributing to some of the findings that are discussed.

We thank the reviewer for their helpful comments. As a result, we have now moved the discussion of the effect of MHC alleles from the discussion into the results section (lines 394-407). In addition, we have also made (the former) Supplementary Figure 5 into a figure in the main text (new Figure 8).

We note the reviewer’s concern about the potential impact of these MHC on the overall conclusions. However, since we see the same pattern of change in post-rebound peak and setpoint viral load in the MHCpos and MHCneg groups, we feel this provides evidence that the overall finding is robust to the effects of MHC on viral control. We have now added a comment to the discussion (line 493-498):

*“It is important to consider potential confounding factors in the study. For example, one factor potentially affecting SIV rebound viral levels is the macaque MHC, and we show that the protective MHC are associated with a lower post-rebound setpoint viral level. Importantly, the same relationship between time of treatment initiation and post rebound peak and setpoint viral levels is observed independently in animals grouped by MHC type, reinforcing that the result is independent of this effect.”*

3. The reservoir/cure field intuits that immune control is operative during ATI and shapes the dynamics of rebound. The authors argue that these findings indicate that approaches to post treatment control should be different for individuals treated early vs treated late. Given that HIV infected individuals rarely are treated within 3 weeks of infection, the main message for humans is that immune dysregulation, exhaustion, viral escape are likely the drivers of relapse post ATI. What new information is being added here that is relevant for next steps in humans?

The reviewer raises an important question of how this work might be best interpreted in the context of HIV infection. We would say that, firstly, the SIV model is being extensively used to model human HIV latency, so understanding the determinants of post-treatment control in SIV is an important goal in itself. However, interpreting the implications of this work for HIV is clearly the ultimate goal of most research in macaques.

We see two important implications of this work. Firstly, a major question for the field is whether a similar relationship between timing of treatment initiation and post-rebound viral dynamics exists in HIV, and if so what the optimal timing of treatment might be? A number of studies have analysed treatment interruption in subjects treated in the first weeks of infection (eg: Colby *et al.*, Rapid HIV RNA rebound after antiretroviral treatment interruption in persons durably suppressed in Fiebig I acute HIV infection. *Nat Med* 10.1038/s41591-018-0026-6 (2018)) and showed rapid viral rebound after early treatment. Our work provides a potential lens through which to understand this work.

Secondly, NHP models of HIV are being used to test interventions aimed at inducing post-treatment control of SIV. Many of these models involve very early anti-retroviral treatment of animals (at 7 to 14 days post-infection). As the reviewer points out, this is very different from the typical treatment timing in HIV. Therefore, consideration of how treatment timing itself effects post-treatment outcome is critical to the correct interpretation of these studies. Given the importance of NHP in preclinical assessment of potential treatments, understanding these differences is critical to rational development of therapeutics.

We have added a paragraph about the relevance of our study to HIV. Lines 439-449

*“Identifying whether the same effects of treatment timing on the dynamics of post-rebound viral levels are seen in HIV is an important future goal. Since the majority of people living with HIV are treated after peak viremia (often significantly later in infection), treatment in HIV is likely more similar to the right-hand side of the curve we observe in SIV. Thus, the major challenge for immunotherapies in HIV may be to reactivate an exhausted immune response rather than stimulating an immune response by providing antigen directly. This contrasts with interventions in early-treated HIV, where boosting of immunity by interventions such as vaccination may be more effective. Since macaque models are an important tool in developing potential therapies, this work suggests that timing of treatment may be an important consideration in extrapolating between SIV and HIV treatment effects.”*

**Reviewer #2:**

Fig 1A: Even though differences between the cohorts are mentioned in the Methods section, it would be helpful to see all viral load trajectories as a supplementary figure, not only selected of cohort 1. Especially study 1d, which has yet to be published, should be illustrated in the supplement (besides all other studies and cohorts). Regarding the cohort-specific results, there appears to be cohort-specific differences. Especially if you pick different cohorts and studies for different analysis. Also, please refer to Fig 1A in the text and explain the data.

We thank the reviewer for these helpful comments. We have now included figures of the viral load trajectories for all cohorts in the main text as Figure 1, so readers can have a clear illustration of the underlying cohorts and data. We note also that the raw data for all cohorts will be available on GitHub upon publication.

Regarding the potential for cohort-specific differences, we believe that the differences between cohorts arose largely because of the different treatment initiation times between cohorts. We also want to emphasise that we did not pick and choose subgroups for analysis. In some cases where we performed an analysis only on a subset of animals, this was because that particular data only existed for that subset. We also note that in some of the previous figures we showed the data grouped by cohort, which we thought might reassure readers that the same patterns are observed in different groups. We have now consolidated the figures to combine the data from different cohorts into a single figure in the main text, and then refer the reader to the supplement for figures broken down by cohort.

We hope this has clarified the analysis.

We have also added the reference to Figure 2A (former Figure 1A) in line 135.

Lines 104 and 105: “shorter periods were used in some animals due to data availability”: This is not clear regarding reproducibility. What is the analyzed time frame? 30 to 60 days or animal-specific? You refer to Table S1, but where is the information on the length of the analyzed time frame?

We apologise for any confusion about the timeframe used to estimate post-rebound setpoint viral levels. The details of which animals used different time intervals was listed in the ‘comments’ column of Supplementary Table 1 in S1 Text and might have been hard to find. This included cohort-specific details of where shorter time intervals were used (because the full time course was unavailable). We have now relabelled this column “Comments / setpoint time intervals” to make this easier to find.

Lines 121 and 129: You state in line 121 that “greatest control is estimated to occur around 18.5 days post-infection”. I do not understand the statement in line 129 that the viral set point is increased if treatment is delayed beyond day 18 post-infection. Both statements seem conflicting. Please explain a little more.

We thank the reviewer for pointing out an inconsistency and lack of clarity as to how we referred to the timing of treatment initiation associated with the best viral control. We note also that this issue was also mentioned in a number of later comments.

Our analysis looked at the pattern of both setpoint and peak post-treatment viral levels. We estimate that the lowest post-rebound setpoint viral levels occur when treatment is initiated on day 18.5. Similarly, the lowest post-rebound peak viral loads are estimated to occur after treatment on day 20.9. However, we note that we do not have experimental data for animals initiating treatment between days 14 and 27, and thus this timing is estimated from the slopes of the data before and after this (so it is not unexpected that these may differ by a few days). In the previous version of the manuscript, we sometimes referred to either day 18 or day 20 as being the nadir timepoint. To clarify this, we now state that we refer to ‘around day 20’ as the optimal time of treatment initiation: lines 192-196

*“Our combined analysis of post-rebound peak and setpoint viral levels suggest that the optimal control of both post-rebound peak and setpoint viral levels is achieved after treatment initiation around days 18-21 days post-infection. For clarity in later discussion we refer to the ‘optimal time of treatment initiation’ as being around day 20 post-infection.”*

Lines 149+: You mentioned that the peak viral load was 0.98 log10 copies per ml higher than set point, regardless of treatment initiation. What does that imply? Can you explain what that means? Additionally, the colors in Supplementary Figure 2 are hard to discriminate. Please use different colors. It is also unclear what models were tested, the goodness of fit, AIC or BICc, and how the parameters differed. Please report those values or values of model comparison. The data looks noisy, and more than one model may fit the data.

We agree with the reviewer that it is interesting that the post-rebound set-point viral level should be consistently around 1 log_10_ lower than the post-rebound peak. There are a number of potentially interesting interpretations regarding immune control of peak versus setpoint viral levels. Alternatively, it may be that availability of susceptible CD4+ T cells is different between peak and setpoint, also affecting viral levels. However, we do not have direct evidence to support these conjectures. Therefore, we now report (lines 187-189):

*“This suggests that similar factors may determine both the post-rebound peak and setpoint viral levels, but with the setpoint being consistently around 1 log10 copies/ml lower than the peak (see Supplementary Figure 4A-C* *in S1 Text)”*

We have also added Supplementary Table 3 in S1 Text with AICc for different models and referenced it in line 180-184. We have also changed the colours of Supplementary Figure 2 in S1 Text to more distinct ones.

Line 174: As mentioned earlier, a supplementary figure showing the viral trajectories of Cohort 2b of all cohorts might be helpful.

As discussed above, we now include the viral load trajectories of all cohorts in the new Figure 1.

Lines 182 to 184: Why split data on day 20? Is that related to the 18.5 days mentioned in line 121 or the 20.9 days in line 145? Did you also try other splits, such as day 7?

As discussed above, we modelled the relationship between timing of treatment initiation and early viral setpoint and peak viral load after treatment. The modelling showed that the best control is predicted to be achieved in animals treated around day 20. Therefore, we aimed to split the data before and after the ‘optimal treatment initiation time’ (which is estimated as day 18.5 and 20.9 for set-point and peak viral load respectively). We refer to day 20 for simplicity. However, we note that since there are no groups that initiated treatment between days 14 and 27, the choice of any day in this interval would not change the groupings.

We did not try other splits such as day 7, as these were not relevant to the analysis.

Lines 191 and 192: You mention a significant association between the timing of treatment and post-rebound set point viral levels. Even though statistically significant, the association of R2 = 0.13 is weak and should be mentioned. You refer to that in lines 201 to 203 that timing of treatment is a major predictor of post-rebound set point viral levels in animals treated before day 20 post-infection. However, this whole analysis could be more convincing, considering that R-square values show relatively weak associations.

We thank the reviewer for suggesting this clarification. The issue here is the large variation in viral loads between individual animals within a cohort. For example, the setpoint viral loads of animals treated on the same day varied between 2 logs and 4 logs. The significance of the correlation (p = 0.0077) suggests that the time of treatment initiation is a significant predictor of setpoint viral level. However, the low R^2^ value arises because it does not explain the individual variation in viral loads (of animals treated on the same day).

We note that for most similar analyses a p-value of 0.0077 would be considered strong (and the R^2^ value would simply not be reported). To clarify this, we have modified the text (line 228-232) to read:

*“timing of treatment was significantly associated with post-rebound setpoint viral levels in the animals treated before day 20 (Figure 3D, p = 0.0077). However, because of the large variation in individual rebound viral loads amongst animals treated on a given day, timing of treatment only explained a small amount of the observed individual viral load variation (adjusted R2 = 0.13).”*

Line 207: You mentioned that R2 = 0.40 is a strong predictor. An R2 = 0.4 is not strong but rather low to average.

We have removed the word ‘strong’.

Line 239 and Fig 3: Why did you only analyze cohort 1 d4, d27 and cohort 3 d60? Why did you not use all the data? You can also use segmented regression combined with a mixed-effect model if linear regression for all data does not work well. Also, what are the group-specific slopes (variability)?

The reviewer refers to our analysis of the effect of duration of treatment (old Figure 3, now Figure 4). These cohorts were analysed because they were the only cohorts where there was a significant diversity in duration of treatment (ie: in other cohorts, all animals in a given study had the same or very similar duration of treatment). Thus, these cohorts provide an opportunity to explore the effects of duration of treatment independent of the timing of treatment initiation.

We have clarified this in the results (line 271-275):

*“In addition to variation in the timing of treatment initiation, several cohorts included animals that initiated ART treatment on the same day post-infection but were treated for different lengths of time before interruption (Supplementary Table 1 in S1 Text). This provides an opportunity to explore the effects of duration of treatment independent of the timing of treatment initiation.”*

Fig 4A to 4D: Again, there seem to be differences between the cohorts affecting the analysis. As mentioned above, seeing the viral load trajectories of all cohorts and treatment groups would be helpful.

We apologise for any confusion. As discussed above, we feel that the differences between cohorts arise largely because of the variation in treatment times. Most importantly, in all cohorts there was a trend to higher DNA / RNA being (somewhat counter-intuitively) associated with lower setpoint viral levels. We have now included all viral load trajectories as Figure 1 (as requested above). In addition, we now show the overall fit to all cohorts for which data was available as the main figure (former Figure 4A-D, currently Figure 5A, B) and include the figures separated by cohort in the supplement, to avoid confusion (Supplementary Figure 5 in S1 Text). We have also added text to lines 301-305 describing new figures.

*“Fitting a linear mixed effect model described by formula (4) (assuming each cohort as a random effect) to this subset of studies, we find a weak but non-significant negative correlation between the level of SIV DNA, SIV RNA just prior to ATI and post-rebound setpoint viral load (Figure 5A, B, Supplementary Table 5* *in S1 Text; we also analysed cohorts separately, see Supplementary Figure 5* *in S1 Text for the results).”*

Line 317 to 319: “animals treated around the optimal time”? Are you referring to the 18.5 days you mentioned earlier? What is the optimal time?

We apologise for the lack of clarity, as discussed above we now define ‘around day 20’ as the optimal time of treatment initiation (lines 192-196) and use this consistent nomenclature throughout.

Fig 6A, B, and C: Do you have any suggestions as to why animals from cohort 1 treated on d4, d6, and d223-377 are poor controllers? Can you give more explanations about what is going on in the figure?
Fig 6D: You do not mention Fig 6D. Please explain Fig 6D.
Fig 6E and 6F: Since you split the data earlier in treatment <= 20 days and > 20 days, you could also perform the “survival analysis” for both treatment groups.

We thank the reviewer for pointing out some confusion related to figure 6. To explain briefly: We find that animals treated very early or very late tend to have higher early set-point control of virus. Animals treated around 2-6 weeks post-infection have the lowest early set-point viral loads. This figure aims to illustrate that this early setpoint viral level only persists for a short time, and then (regardless of treatment time) the majority of animals reach viral levels >10,000 copies / ml by 4 months post-infection.

To illustrate this better we have removed the old panel 6D and replaced it with a figure showing the survival curves divided into ‘animals treated on day 14-43’, versus those treated earlier or later (currently Figure 7A) and modified the text accordingly in lines 354-366. This better illustrates the short-term benefits of treatment at the optimal time, as well as the loss of this effect over time. We include the per-cohort survival times in the supplement for completeness.

Lines 397 to 400: “overall we observed around 30% of macaques showed early post-rebound control”. Where is that number coming from? You talked about 20% controllers, which seems to be a background level of long-term control. Please explain where the 30% is coming from. Moreover, “the proportion of early controllers was higher in animals treated in a window between day 15 - 41 post-rebound”? I do not understand that sentence. Is days of treatment 15 to 60? And also seems to conflict with the result earlier in line 121 that “greatest control is estimated to occur around 18.5 days post-infection” (or between days 14 and 27 in line 114). Can you explain how this all fits together?

We thank the reviewer for pointing out this confusion. Referring to the entire study, around 30% of macaques have maintained viral levels <10,000 copies / ml by 60 days post-rebound. However, we also wanted to show that for animals treated around the optimal treatment time, this proportion of animals with this early control is higher. However, this early control is only temporary, since by day 150 the proportion of animals with viral levels <10,000 copies is similar regardless of the timing of treatment initiation. We have now added a clarifying comment (lines 457-465);

*“**That is, overall we observed around 30% of all macaques in our 3 cohorts showed post-rebound control of plasma viral load to below 10,000 copies ml-1 between days 30-60 post-rebound. The proportion of animals showing control at this time was higher in animals treated in a window around the optimal treatment time (54% of animals treated between day 14-43 post-rebound showed control to <10,000 copies /ml out to day 60(Figure 7A)). However, this higher level of early control is only temporary. By 150 days after rebound the proportion of animals with viral levels <10,000 copies / ml was similar between animals initiating treatment at different times (Figure 7A). ”*

Supplementary Figure 5: It is interesting that different models work for A and B, where there is a difference between the MHC alleles in set point VL (A) but no difference in peak VL (B). The data seems equally distributed. Visually, there also appears to be no difference in A. Did you test models that differ in multiple parameters (not only b0)? Please report goodness of fit, AIC, or BICc for model comparison (or other values of model comparison) for both set point and peak VL and the models you tested. Additionally, it is tough to gather the parameter information since they are in different tables and mixed with different results. Please make it easier for the reader to understand your results.

Our analysis shows that the initial viral load (*b0*) was the major parameter that was impacted by macaque MHC type in our modelling of setpoint viral levels, whereas MHC type had no significant effect on peak post-treatment viral levels. We fitted the model to setpoint and peak viral load data allowing different parameters to vary between datasets with and without protective alleles (see table below). As shown below, for the post-rebound setpoint viral level, the model with the different b_0_ has the smallest AICc, while the variation of parameters in the model fitted to peak viral load data does not lead to a significant variation of AICc. We have now added this table to the supplement (Supplementary Table 7 in S1 Text) that shows the fitting for all combinations of varying one and two parameters and the corresponding AICc and text at lines 397 – 402.

*“To investigate the potential effects of MHC alleles on our results, we separated animals with and without these protective alleles and repeated our analysis of the relationship between time of treatment initiation and post-rebound peak and setpoint viral levels. We again compared models where we allowed different parameters to vary between the groups (model goodness of fits by AICc is summarised in Supplementary Table 7* *in S1 Text).”*

| **Number of parameters that differ between datasets** | **Parameters that are different for datasets with the presence or absence of protective MHC-class 1** | **AICc. Setpoint data** | **AICc. Peak data** |
| --- | --- | --- | --- |
| 1 | *b_0_* | -6.716 | -91.46 |
| 1 | *b_1_* | 1.486 | -92.68 |
| 1 | *k* | 1.683 | -91.21 |
| 1 | *δ* | 2.978 | -91.21 |
| 1 | *t_min_* | 6.46 | -91.25 |
| 2 | *b_0_, t_min_* | -4.948 | -89.56 |
| 2 | *b_0_, δ* | -4.576 | -89.33 |
| 2 | *b_0_, b_1_* | -4.581 | -90.64 |
| 2 | *b_0_, k* | -4.877 | -89.32 |
| 2 | *b_1_, t_min_* | 5.138 | -91.11 |
| 2 | *b_1_, δ* | 1.994 | -90.56 |
| 2 | *b_1_, k* | 2.003 | -90.67 |
| 2 | *δ , t_min_* | 5.056 | -89.26 |
| 2 | *δ, k* | 3.792 | -89.04 |
| 2 | *k, t_min_* | 0.1138 | -90.17 |
| 0 | All parameters are the same | 5.927 | -93.34 |
| 5 | All parameters are different | -4.48 | -85.73 |

**Part III – Minor Issues: Editorial and Data Presentation Modifications**

**Reviewer #1:**

1. This statement in the legend of Figure 1 is not clear: “The duration of treatment for the setpoint and peak viral load at primary infection (Prim) is assumed to equals 0 days.”

We apologise for the confusing wording. We have modified this to read (Figure 2 currently, line 729-731): “*When fitting model (2), we assume that the duration of treatment for the setpoint and peak viral load at primary infection (Primary) equals to 0 days.*

2. It should be noted that for animals mucosally inoculated, the time for actual infection could be less clear than indicated in Figure 1.

The reviewer correctly points out that there might be slight differences in the early kinetics of virus between mucosal infection and intravenous infection, and thus comparing timing of treatment initiation may be problematic. However, there is only one group that is infected mucosally, and these animals were treated on days 223-377 (see Supplementary Table 1 in S1 Text). Therefore, although this is a theoretical issue, it seems unlikely that minor differences in early viral kinetics would affect our conclusions. We have added a short note (line 498-502):

“*Another factor is that some monkeys were infected intrarectally, and mucosal infection may affect early viral kinetics compared with IV infection. However, the only group with mucosal infection initiated treatment on days 223-377 (Supplementary Table 1* *in S1 Text), and it seems unlikely that this minor differences in early viral kinetics would affect our conclusion.”*

3. Line 688: “Vertical dashed lines indicate days of treatment” should be stated, “… days of treatment initiation.”

We have clarified this as requested to read: “*Vertical dashed lines indicate the timing of antiretroviral treatment initiation*” (line 724).

**Reviewer #2:**

- refer to Fig 1A
- In line 77 you mention the “Fiebig 1” stage: Not all readers, especially those outside HIV, may be familiar with the Fiebig stages. Please add one sentence for the Fiebig I stage or add a reference

We have added citation to line 109:

*E. W. Fiebig et al., Dynamics of HIV viremia and antibody seroconversion in plasma donors: implications for diagnosis and staging of primary HIV infection. AIDS* ***17****, 1871-1879 (2003).*

- line 316: copies per ml

We have corrected to copies/ml.

- Line 517 and 518: Specify the limit of detection

We have specified the limit of detection of each group in the Supplementary Table 1 in S1 Text.
