# Supplementary material for: Timing of initiation of anti-retroviral therapy predicts post-treatment control of SIV replication
Source: PLoS Pathog. 2023 Oct 6;19(10):e1011660. doi: 10.1371/journal.ppat.1011660 (PMC10558076; doi:10.1371/journal.ppat.1011660)
Supplement: Attachment — Submitted filename: Response to Reviewers-rev 2 2023 08 08 .docx [file ppat.1011660.s003.docx]

Reviewer Comments (if any, and for reference):

Reviewer's Responses to Questions

Part I - Summary

Please use this section to discuss strengths/weaknesses of study, novelty/significance, general execution and scholarship.

Reviewer #1: Pinkevych and coworkers have provided detailed and helpful responses to the criticisms and suggestions of both reviewers, resulting in a much improved manuscript. Their study of SIV-infected and ART-suppressed macaques is novel in analyzing data from 10 different animal cohorts to address correlates of control following discontinuation of antiretroviral therapy. As they note, NHP models offer advantages for addressing viral dynamics post ATI in that set points following rebound cannot be established in humans given medical and ethical issues for ART to be reinitiated. The key points of the paper are well stated, namely that 1) when treatment was given before 3 weeks of infection, the longer the time to initiate treatment, the lower the post ATI peak and set point; and 2) when treatment was started after 3 weeks of infection, the longer time to initiate treatment correlated with higher post-ATI set points. These interesting patterns seen across multiple cohorts suggest different underlying mechanisms for viral control following early vs. late treatment, and reasonable hypotheses are proposed. The impact of MHC alleles on these findings is also started more clearly, and it is impressive that these same patterns were seen even when controlling alleles were grouped. There are also many somewhat surprising findings including that 1) plasma viral loads prior to treatment were not predictive of post-rebound kinetics; and 2) larger reservoir size did not directly influence high rebound viral levels.

Overall, the manuscript has been improved in clarity and in driving home the importance of these findings in considering mechanisms that could be relevant for control of HIV post-ATI.

I have only minor issues for them to address.

Reviewer #2: The authors have revised the manuscript and followed my suggestions. I appreciate your work and I recommend this manuscript to be published in this journal.

Part II – Major Issues: Key Experiments Required for Acceptance

Please use this section to detail the key new experiments or modifications of existing experiments that should be absolutely required to validate study conclusions.

Generally, there should be no more than 3 such required experiments or major modifications for a "Major Revision" recommendation. If more than 3 experiments are necessary to validate the study conclusions, then you are encouraged to recommend "Reject".

Reviewer #1: (No Response)

Reviewer #2: (No Response)

**Part III – Minor Issues: Editorial and Data Presentation Modifications**

**Please use this section for editorial suggestions as well as relatively minor modifications of existing data that would enhance clarity.**

Reviewer #1: 1. I did found it helpful to have the new summary table listing correlations (or lack thereof) as Supplemental Table 8. This provides an anchor for the reader to take the breadth of diverse findings that were presented throughout the Results. However, there is data presented in Results Section that does not refer to Table 8. In fact, Suppl. Table 8 is only cited once in the manuscript (Line 221). The authors should cite Suppl Table 8 wherever appropriate, when data summarized in this Table are described in the Results Section.

For example, Line 247 (“viral load at treatment was a predictor of setpoint viral load during rebound (adjusted R2 = 0.40, p <0.0001) (Figure 3E) and Line 255 (“ … found that this was weakly, but significantly, associated (adjusted R2 = 0.11, p=0.0024) (Figure 3F”) should refer to Suppl. Table 8.

There are also points made in Results that should be added to this Summary Table: Line 763 Figure 5- “… no relationship between SIV DNA and RNA with the setpoint viral load we used. Fixed effect slopes are -0.066 and -0.2015763, p-values for slopes are 0.56 and 0.117 for SIV” ; and Line 315- “We observed no significant difference between time-to-detection of plasma SIV in animals with higher or lower than median post-treatment setpoint viral levels, suggesting that time-to-rebound is not associated with post-rebound setpoint viral levels (Figure 5D-F)”

Also, for clarity, the language of this Table should fit exactly the language in the text (i.e. change “viral load at treatment” in Suppl Table 8 to “viral load at treatment initiation”; change “primary peak viral load” in the Table to “peak viral load in primary infection.”)

*We thank the reviewer for suggesting these changes to improve the clarity of the manuscript. We have now cited Suppl. Table S8 in the main text as suggested, added additional details to the supplementary table, and made the language consistent between text and table.*

2. The authors use a rather generous definition of post rebound "control" with a set point of <10,000 copies/ml, seen in 20% of monkeys maintained on long term viral control. They note that this is rarely observed in untreated primary SIVmac239 infection. Were there any animals with more impressive levels of control (e.g. <1,000 copies/ml)? If so, it would be interesting to note if there was anything special about such animals even if the numbers (of animals) did not rise to a level of statistical significance.

*We have now prepared a matching figure using the viral load cutoff of 1000 copies (Supplementary Figure 6, panels D, E, F). The same overall pattern is seen as with the threshold of 10,000 copies. However, in some cases this is no longer significant due to the lower number of animals with control <1000 copies. We did not identify any special features of animals with more impressive viral control.*

3. In the Discussion, Line 513 states: “In our previous studies we have investigated how timing of treatment can affect the frequency of rebound from latency.” Please list the reference to which you are referring.

*We have now added a reference as suggested.*

Reviewer #2: (No Response)

PLOS authors have the option to publish the peer review history of their article (what does this mean?). If published, this will include your full peer review and any attached files.

If you choose “no”, your identity will remain anonymous but your review may still be made public.

Do you want your identity to be public for this peer review? For information about this choice, including consent withdrawal, please see our Privacy Policy.

Reviewer #1: No

Reviewer #2: No
